# Internet search and medicaid prescription drug data as predictors of opioid emergency department visits

**DOI:** 10.1038/s41746-021-00392-w

**Published:** 2021-02-11

**Authors:** Sean D. Young, Qingpeng Zhang, Jiandong Zhou, Rosalie Liccardo Pacula

**Affiliations:** 1 Department of Emergency Medicine, University of California, Irvine, CA USA; 2 University of California Institute for Prediction Technology, Department of Informatics, University of California, Irvine, CA USA; 3 School of Data Science, City University of Hong Kong, Kowloon, Hong Kong SAR China; 4 The Sol Price School of Public Policy and Leonard D. Schaeffer Center for Health Policy & Economics, University of Southern California, Los Angeles, CA USA

**Keywords:** Health policy, Risk factors

## Abstract

The primary contributors to the opioid crisis continue to rapidly evolve both geographically and temporally, hampering the ability to halt the growing epidemic. To address this issue, we evaluated whether integration of near real-time social/behavioral (i.e., Google Trends) and traditional health care (i.e., Medicaid prescription drug utilization) data might predict geographic and longitudinal trends in opioid-related Emergency Department (ED) visits. From January 2005 through December 2015, we collected quarterly State Drug Utilization Data; opioid-related internet search terms/phrases; and opioid-related ED visit data. Modeling was conducted using least absolute shrinkage and selection operator (LASSO) regression prediction. Models combining Google and Medicaid variables were a better fit and more accurate (*R*^2^ values from 0.913 to 0.960, across states) than models using either data source alone. The combined model predicted sharp and state-specific changes in ED visits during the post 2013 transition from heroin to fentanyl. Models integrating internet search and drug utilization data might inform policy efforts about regional medical treatment preferences and needs.

## Introduction

Opioid misuse currently kills 130 Americans per day, making it a top public health concern in the United States^1^. Rates of opioid-related morbidity and mortality continue to increase, requiring new tools and approaches to prevent overdose. For example, there have been consistent year-over-year increases in predictors of mortality, such as the number of opioid-involved emergency department visits^2,3^, and 911 calls requiring the use of naloxone or multiple naloxone administrations^4^. The epidemic is also rapidly evolving: opioid analgesics were the primary cause of overdose until 2010, but heroin (subsequently) and fentanyl (currently) have been the primary drivers of recent opioid mortality rates^5^.

Local community health care providers, first responders, and public safety systems, which are particularly impacted by the crisis, are desperate for higher quality and more real-time data to monitor the problem and intervene in a timely manner. Timely information transmission is also essential for emergency medical service providers to be better prepared for a patient’s arrival and to prevent mortality^6,7^. However, there are a number of problems with current data, including lack of access to real-time surveillance at the local level^8^; 1-year lag times in the release of data on mortality, opioid prescribing, and emergency department (ED) visits^9^; and lack of data on reasons for regional and temporal differences in risk behaviors and mortality^10^. Taken together, new data sources and tools are needed to better monitor and predict opioid-related outcomes to save lives.

Integrating online social/behavioral data, obtained in near real-time, may help overcome some of the issues associated with traditional public health data. For example, internet search data from Google have already been found to be associated with and/or predictive of a number of health-related outcomes, including HIV^11^, heroin-related emergency department visits^12^, suicide^13^, cardiovascular disease^14^, and syphilis^15^. Importantly, internet search data can typically be broken down by Designated Market Areas (DMA), allowing analyses to inform regional differences in risk behaviors and mortality. However, there are limitations of previous studies using internet search data to predict health outcomes. For example, previous studies using internet search data to predict opioid-related outcomes (e.g., emergency department visits for heroin) have used internet search data as the only predictor. Compared to internet searches for opioids, which are indirectly linked to opioid-related outcomes, more directly linked (medical data) sources, such as prescription drug data, might be more accurate predictors of opioid outcomes. In addition, previous work focusing on using internet search data to predict opioid-related outcomes focused on a small number of cities, limiting the generalization of this research. The prior research was also only studied up through 2011^12^, limiting the ability to learn whether the models would be able to predict the sharp increase in opioid overdoses after 2011 that resulted from the increased use of fentanyl.

There are also obvious limitations with internet search data: it is unclear whether individuals who are searching online for opioid-related information would act on those searches, search data by itself may not provide enough information to inform interventions, and selectivity bias influences predictive ability. Nonetheless, studies on internet search data have found high correlations between searches and public health outcomes at the local level. It may be possible to harness their contribution and overcome their limitations by combining them with clinical data, which has not yet been done.

In this study, we seek to assess whether combining internet search and prescription drug data generate improved predictions of emergency department (ED) visits, including predicting geographic and longitudinal trends in opioid-related ED visits. We attempt to predict opioid-related ED visits because they occur more frequently (provide more data) than opioid-related deaths, are strongly associated with opioid-related deaths^2,16^, and because the effective distribution of naloxone has led to a reduction in the number of opioid related deaths while rates of ED visits remain high^17^. We report on the results of the models as well as potential interpretations of the qualitative results (i.e., the specific Google searches and drugs most commonly prescribed) across geographic regions.

## Results

### Statistical results

**Fig. 1:**
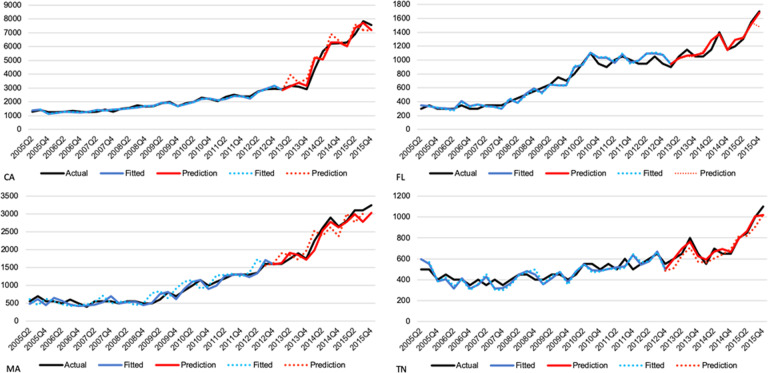
Results. One-quarter-ahead (solid) and two-quarter-ahead (dashed) predictions of the ED visits for four typical states based on Google search and drug use data.

Based on *R*^2^ and RMSE criteria, the most accurate and best-fitting models predicting one-quarters or two-quarters-ahead for every state combined both the Google and Medicaid variables in the same model (Fig. 1 and Table 1). We can see in Fig. 1 of four different states, that the model was able to predict opioid-related ED visits with high accuracy based on Google search and drug use data with one-quarter-ahead (solid) and two-quarter-ahead (dashed) models. The values for the *R*^2^ from these models (which range from 0.913 to 0.960 across states in the one-quarter-ahead prediction) are consistently higher than models using either set of data alone, while the RMSE values (which range from 13.48 to 361.96) are consistently lower. Although models using Google data or Medicaid data alone performed reasonably well, the one-quarter-ahead prediction models with Medicaid data alone performed better (in terms of higher *R*^2^ and lower RMSE) than the same prediction model using just Google data for all states, except two (Georgia and Minnesota). The same was true for the two-quarter-ahead prediction models, although the two states where the Google data prediction models outperformed the Medicaid data were different (Indiana and Massachusetts). Neither outperformed the model using both types of data, particularly in terms of RMSE. Moreover, our combined model was able to predict sharp changes in opioid-related ED visits tied to changes in the primary drivers of the opioid crisis from heroin to fentanyl post 2013. The ability to accurately predict these shifts represents a major benefit of combining these data.

**Table 1:** One-quarter-ahead (and two quarter-ahead) prediction accuracy of different models across states, 2005–2015.

	Google search data		SDUD Rx utilization data		Both Google and drug data	
	*R*^2^	RMSE	*R*^2^	RMSE	*R*^2^	RMSE
CA	0.8736 (0.7844)	638.6979 (834.2924)	0.9062 (0.8282)	550.1519 (744.5870)	0.9594 (0.8969)	361.9566 (576.9079)
FL	0.8059 (0.7200)	97.0510 (116.5707)	0.8489 (0.7370)	85.6269 (112.9681)	0.9271 (0.8375)	59.4883 (88.7939)
GA	0.8697 (0.6800)	16.4743 (25.8185)	0.8409 (0.7387)	18.2066 (23.3316)	0.9127 (0.8278)	13.4825 (18.9402)
IN	0.856 (0.8127)	58.8990 (67.1762)	0.8851 (0.7547)	52.6304 (76.8859)	0.9405 (0.8503)	37.8547 (60.0672)
MA	0.8522 (0.7560)	219.4779 (282.0361)	0.8885 (0.7022)	190.6938 (311.6135)	0.9287 (0.8157)	152.4159 (245.1396)
MN	0.899 (0.7672)	51.6804 (78.4571)	0.8208 (0.7865)	68.8408 (75.1380)	0.9602 (0.8420)	32.4432 (64.6257)
MO	0.8559 (0.7295)	51.0897 (69.9864)	0.8812 (0.7628)	46.3891 (65.5383)	0.9271 (0.8207)	36.338 (56.9789)
NJ	0.8668 (0.7651)	254.2480 (337.6893)	0.9013 (0.7835)	218.8394 (324.1491)	0.9555 (0.8599)	147.0135 (260.8051)
NY	0.7541 (0.7249)	430.4102 (455.1811)	0.8900 (0.7231)	308.49 (489.5146776)	0.9462 (0.8077)	215.769 (407.9654628)
TN	0.8566 (0.7458)	60.3684 (80.3787)	0.8946 (0.7872)	51.7539 (73.5375)	0.9471 (0.8345)	36.6575 (64.8513)
WI	0.8005 (0.7520)	153.2267 (170.8507)	0.8993 (0.7620)	108.8656 (167.3505)	0.9554 (0.8218)	72.4859 (144.8327)

For robustness, we also compared the performance of the LASSO-based prediction model for each state with a mixed model and pooling analysis model (one combined model for all states). Random effects in the mixed model accounted for individual state differences in prescribed drugs. The pooling analysis model was used to provide an overall summary by combining subgroup state data. Results suggest that the LASSO prediction model on individual states outperforms the mixed model and pooling analysis model (supplementary materials). We also identified differences in the most frequently searched internet terms and prescribed medications across states (Table 2). The predictive strengths of the search items and drugs differed among states. For instance, oxycodone occurs to be one of the top three most predictive drugs in the states of GA, IN, MA, MN, NJ, and NY, while fentanyl and opioid (or non-opioid) ranks among the five most predictive search items in states of CA, GA, IN, MA, MN, NJ, NY, and WI. We can see the varying predictive strength of the search term, naloxone, which is of great importance for efficient decision-making of early interventions, since the effective distribution of naloxone has led to a reduction in the number of opioid related deaths while rates of ED visits remain high. For each state, the prediction model that incorporated both the social and medical data together performed.

**Table 2:** Opioid-related prescribed drugs and Google search terms for each state.

CA		FL		GA	
Top drugs	Top search terms	Top drugs	Top search terms	Top drugs	Top Search Terms
LEVOCARNIT	Fentanyl	LIDOCAINE-	Fentanyl	OXYCODONE	opioid conversion
NOVOLOG PE	Opioid withdrawal	TRAMADOL T	What is opioid	LEVOCARNIT	opioid
TRAMADOL T	Non-opioid	GUANFACINE	Opioid abuse	PHOSPHA 25	suboxone
BACLOFEN T	Methadone	GENOTROPIN	Morphine	KENALOG-40	opiates
OXYCODONE	Symptoms	ERBITUX	Oxycodone	TRAMADOL H	fentanyl
INFED IRO	Narcotic	BACITRACIN	Opiates	NOVOLOG PE	opioid medications
ERBITUX	Opioid abuse	OXYCODONE	Heroin	VENTOLIN H	non opioid
OXYCODONE	Opiates	MORPHINE	Suboxone	ASPIRIN 5G	hydrocodone
TRAMADOL H	Tramadol	MAGNESIUM	Opioid	BACLOFEN T	methadone
MORPHINE	What is opioid	TRAMADOL H	Opioid antagonist	ASPIRIN LO	morphine
IN		MA		MN	
Top drugs	Top search terms	Top drugs	Top search terms	Top drugs	Top Search Terms
OXYCODONE	What is opioid	AMMONIUM L	Non-opioid	TRAMADOL T	non opioid
TRAMADOL H	Oxycodone	OXYCODONE	Opioid withdrawal	OXYCODONE	oxycodone
LEVOCARNIT	Fentanyl	CALCIUM 50	Fentanyl	NORETHINDR	opioid withdrawal
AZITHROMYC	Heroin	EPIPEN JR	Morphine	BACLOFEN T	fentanyl
KENALOG-40	Non-opioid	CARAFATE 1	Heroin	NOVOLOG PE	oxycodone
MAGNESIUM	Opioid withdrawal	CIPROFLOXA	Oxycodone	CALCIUM 50	morphine
CIPROFLOXA	Opioid abuse	GENOTROPIN	Narcotic	ASPIRIN LO	methadone
TRAMADOL T	Morphine	TRAMADOL H	Opioid abuse	KENALOG-40	narcotic
ASPIRIN 5G	Opiates	MORPHINE	Hydrocodone	TRUVADA 20	opioid abuse
CLINDAMYCI	Methadone	LEVOCARNIT	Suboxone	PREDNISOLO	hydrocodone
MO		NJ		NY	
Top Drugs	Top search terms	Top drugs	Top search terms	Top drugs	Top Search Terms
TRAMADOL 5	Tramadol	TRAMADOL T	Opiates	CLINDAMYCI	non opioid
LIQUITEARS	Opiates	OXYCODONE	Opioid	CHLORZOXAZ	fentanyl
ETHOSUXIMI	Opioid	BUSPIRONE1	Fentanyl	OXYCODONE	opioid withdrawal
FLUTICASON	Opioid withdrawal	DOCUSATE S	Opioid withdrawal	BICILLIN L	opioid
TRAMADOL T	Narcotic	MORPHINE	Non-opioid	TRAMADOL T	morphine
CALCIUM 50	Fentanyl	EPIPEN JR	Morphine	BUTALBITAL	suboxone
LEVOCARNIT	Suboxone	ASPIRIN 5G	Suboxone	LEVOCARNIT	hydrocodone
MORPHINE	Morphine	KENALOG-40	Morphine	MAGNESIUM	narcotic
ASPIRIN LO	Hydrocodone	METRONIDAZ	Hydrocodone	BACLOFEN T	oxycodone
NICOTINE 2	Oxycodone	PHOSPHA 25	Narcotic	CLINDAMYCI	tramadol
TN		WI			
Top Drugs	Top search terms	Top drugs	Top search terms		
ERYPED 200	Opioid	TRAMADOL H	Fentanyl		
TRAMADOL H	Tramadol	ASPIRIN 5G	Opioid withdrawal		
MORPHINE	Opioid conversion	EPIPEN EPI	Non-opioid		
CLINDAMYCI	Morphine	MORPHINE	Morphine		
KENALOG-40	Opioid withdrawal	ARTIFICIAL	Opiates		
EPIPEN JR	Non-opioid	EPIPEN JR.	Narcotic		
LEVOCARNIT	Narcotic	ANTIPYRINE	Hydrocodone		
MAGNESIUM	Fentanyl	OXYCODONE	Opioid medications		
OXYCODONE	Hydrocodone	LEVOCARNIT	Oxycodone		
TRAMADOL T	Oxycodone	10% PREMAS	Tramadol		

## Discussion

Findings underscore the need for public health agencies to integrate novel and diverse data sources and methods (e.g., combining near real-time internet search data along with traditional health care data) into their monitoring and surveillance efforts. Agencies need as much information as possible to prepare for the impact of the constantly evolving opioid epidemic. Models that incorporate social and medical data together may better prepare hospitals and health systems for the changing needs of the opioid crisis. For example, similar models could be developed to identify geographic areas likely to experience increases and/or rapid changes in the need for treatment services in response to fentanyl cases. They could also provide insights into interventions among areas with particularly high rates of HIV-related stigma or unrecognized HIV infection that might be linked to substance use^18,19^. Health departments could use these forecasts to improve linkages between hospital emergency departments and treatment providers with unused capacity, such as buprenorphine waivered doctors who are treating fewer patients than allowed by their waiver.

There are a number of more specific implications of this research. First, contrary to possible intuition, Medicaid data does not appear to be a definitively better predictor of opioid-related visits than internet search data. In fact, the model incorporating both internet search and Medicaid data together demonstrated the best performance. This is important information to motivate researchers to explore the use and integration of internet search and other social data sources into modeling efforts. The results also suggest, that, for complicated issues such as the opioid crisis, models combining diverse sources of data might be better at predicting health outcomes compared to just one source of data. Second, the proposed model was able to predict longitudinal changes in opioid-related ED visits, even in years such as 2013 where traditional health econometric models have typically not performed well. This again suggests the importance and potential of integrating social/behavioral data (e.g., internet search) along with traditional medical (e.g., prescription drug) data in epidemiological efforts. Finally, this work suggests that public health agencies should explore integrating these novel data sources and modeling methods into their opioid-related surveillance efforts.

A further advantage of this modeling approach is that it allowed us to flexibly identify different key predictors of ED visits by geographic area, and confirmed that key predictors differ across states and over time. For example, while suboxone was a commonly used search term across most states, methadone was a more common and important search term in Minnesota and Indiana. This further highlights the need to make use of modeling tools and data that accurately reflect the local experience^10^.

This study has limitations, primarily related to the data sources. We were limited by the ability to collect observational rather than individual-level data; to acquire quarterly data rather than more frequently updated data; and by only being able to include 11 states at the aggregate state level, rather than a larger number of states with the ability for finer-grained within state analysis.

Even with these limitations, findings from this study clearly demonstrate that the integration of social/behavioral and medical information is more powerful for predicting geographic and temporal changes in opioid-related ED visits than either source of information alone. Although an increasing number of studies have used social media and/or internet search data to predict public health outcomes, an ongoing criticism of such studies is that they are an indirect and possibly biased source of behavioral health information and hence will have little predictive value compared to more directly linked medical data. This analysis suggests that this may not always be the case, as our models using internet search variables alone did reasonably well predicting ED visits and even outperformed models using only medical data in a small number of states. However, the predictive power of the models combining both data sources is clearly better.

Overall, results suggest that the integration of social/behavioral data, which are often available in near real-time, combined with traditional public health data, may improve surveillance efforts compared to current methods using traditional public health data alone. Although too early to directly implement into interventions, these types of data and approaches might be further studied and used to uncover the regional trends in preferences and/or interest in different types of medication assisted therapy to help geographically target educational campaigns and interventions to regions most in need and accepting of that treatment.

## Methods

### Data sources and methods

From January 1, 2005 to Dec 31, 2015, we collected quarterly Google Trends data for 22 commonly used opioid-related internet search terms and phrases for all states from 2005 through 2015. The terms include opioid medications (e.g., fentanyl and hydrocodone), opioid recreational drugs (e.g., heroin), and general searches about opioids and overdose risk. The Google Trends index provides the normalized search frequency based on the relative search volume of searched keywords at a specific time. The full list of the keywords, adapted from a previous study using opioid-related search terms^12^, is presented in Table 3. Although the search term variables/data are slightly different than the earlier study because they include additional years of data, they were picked because that study had already shown the relationship between opioid-related search terms. We sought to reuse the terms that had already been found associated with heroin; however, we also included a small number of additional terms by using the google trends tool to find search terms related to those initial terms. The Google data retrieval was done on Oct 10, 2018. We chose the period of data for study as it is one where there were substantial shifts in the drivers of opioid-related mortality (e.g., from prescription drug use to heroin to fentanyl) that we want our model to capture and because the data were publicly available.

**Table 3:** The 22 opioid-related terms used to collect the Google Trends data, January 1, 2005 to Dec 31, 2015.

Fentanyl	Narcotic	Opioid analgesics	Opioid side effects
Heroin	Non opioid	Opioid antagonist	Opioid withdrawal
Hydrocodone	Opiates	Opioid conversion	Oxycodone
Methadone	Opioid	Opioid definition	Suboxone
Morphine	Opioid abuse	Opioid medications	Symptoms
Tramadol	What is opioid		

We obtained state-level quarterly drug utilization data during the same time period from the State Drug Utilization Data (SDUD), provided by Medicaid.gov^20^. These data represent medical prescriptions filled on an outpatient-basis and paid for by state Medicaid agencies. We included the eleven states with complete data for each year (California, Florida, Georgia, Indiana, Maryland, Minnesota, Missouri, New Jersey, New York, and Tennessee, Wisconsin) in the final analysis. For each of these 11 states, for each quarter, we identified the 100 prescribed drugs (based on the National Drug Code (NDC)) most correlated with relative Google search volume of opioid-related keywords. The prescriptions included both opioids and non-opioid drugs. We also collected quarterly data on opioid-related emergency department (ED) visits from the Healthcare Cost and Utilization Project (HCUP), Fast Facts data on opioid-related hospital use^21^. HCUP ED data were collected starting and ending one quarter later than the Google data (April 1, 2005 to March 31, 2016), as the analysis was designed to predict number of opioid-related ED visits. This study was waived from review by the UCLA institutional review board (IRB) as data are anonymous and reported aggregately.

### Data analysis

The study was designed to determine the best fitting model incorporating internet search and/or drug utilization data as predictors of opioid-related ED visits. To model the number of ED visits, which are count data, we used the negative binomial generalized linear model (nbGLM), a widely adopted statistical model for count data that has been frequently used in public health prediction models^11,13,22–24^. We adopted the Least Absolute Shrinkage and Selection Operator (LASSO) approach^25^ to identify the subset of predictors that have the best predictive power among the list of search keywords, and 100 most frequently used drugs for each state. We validate the LASSO models by performing a retrospective out-of-sample prediction experiment, in which we use one set of data (historical data) to train the parameters of the models, and then use the trained model to predict the ED visits in another set of data (future event). More specifically, we validate the models’ efficacy in performing one-quarter-ahead and two-quarter-ahead prediction tasks. We also compared the performance of the LASSO-based prediction model for each state with a mixed model and pooling analysis model (one combined model for all states). Comparative results suggest that the LASSO prediction model on individual states outperforms the mixed model and pooling analysis model (supplementary materials). In addition, the LASSO method is effective in the minimization of prediction errors that are common in statistical models to optimally select the search items that are predictive with high accuracy. The accuracy of LASSO method and low sensitivity to parameters are the result of its advantages to include shrinkage of coefficients. This approach is used to reduce variance and minimizes bias to ensure the validation of the predictions with an out-of-sample 10-fold cross validation approach. The prediction performance of LASSO is provided in Table 1, based on *R*^2^ and RMSE evaluation criteria. The most accurate and best-fitting models predicting one-quarters or two-quarters-ahead for every state combined both the Google and Medicaid variables in the same model (Fig. 1). Further, to verify the sensitivity of the prediction model based on the search terms chosen, we conducted both forward and backward stepwise regression (Supplementary Table 5 and Supplementary Table 6). The superior performance of models integrating both internet search and Medicaid data highlights the importance of combining the two datasets for better performance. It suggests that internet searches for opioids is associated with actual opioid use/outcomes. Details of the statistical models/approach are presented in the supplementary materials.

To evaluate the accuracy of the proposed models and identify the most predictive search terms for each state, we considered the commonly used *R*^2^ (*R*^2^) and Root Mean Square Error (RMSE) statistics in an out of-sample 10-fold cross validation approach. *R*^2^ measures the extent the variance of predictors explains the variance of the response. A larger *R*^2^ value indicates a model with greater explanatory power. RMSE is the standard deviation of the prediction errors. A smaller RMSE indicates a more accurate model. To address changing trends in opioid-related online search terms, the search terms in the model were updated each year. We performed experiments for two ED visit prediction scenarios: (i) one-quarter-ahead prediction, and (ii) two-quarter-ahead prediction. We compared the prediction performance for the two scenarios in each state. The proposed model performed well for both scenarios, with the prediction accuracy for one-quarter-ahead prediction being higher than for two-quarter-ahead prediction.

### Reporting summary

Further information on experimental design is available in the Nature Research Reporting Summary linked to this paper.

## Supplementary information

Supplemental Information

Reporting Summary

## Data Availability

The data used in this analysis are publicly available online through Medicaid and HCUP. Google data may be available upon request, pending confirmation from Google.

## Code availability

Code for analyses may be available upon request, pending approval from the IRB. Data were analyzed using python. Supplementary materials are provided to assist in replication by describing the complete models and math used for analysis.
