# Supplementary material for: Internet search and medicaid prescription drug data as predictors of opioid emergency department visits
Source: NPJ Digit Med. 2021 Feb 11;4:21. doi: 10.1038/s41746-021-00392-w (PMC7878816; doi:10.1038/s41746-021-00392-w)
Supplement: Supplementary file 1 — Supplemental Information [file 41746_2021_392_MOESM1_ESM.pdf]

Supplementary Materials for

Internet Search and Medicaid Prescription Drug Data as Predictors of Opioid Emergency Department Visits

This PDF file includes:

Materials and Methods
Supplementary Fig. 1
Supplementary Fig. 2
Supplementary Fig. 3
Supplementary Fig. 4
Supplementary Fig. 5
Supplementary Fig. 6
Supplementary Fig. 7
Supplementary Table 1
Supplementary Table 2
Supplementary Table 3
Supplementary Table 4
Supplementary Table 5
Supplementary Table 6

Materials and Methods

Data

From January 1, 2005 to Dec 31, 2015, we collected quarterly Google Trends data for 22 opioid-related search terms and phrases (shown in Table S1) for all states from 2005 through 2015. The terms include opioid medications (e.g. fentanyl and hydrocodone), illicit opioids (e.g. heroin), and general searches about opioids (e.g. "what is opioid?"). The Google data retrieval was done on Oct 10, 2018.

We obtained state-level quarterly drug utilization data during the same time period from Medicaid.gov, which reports information on prescription drug fills reported to and paid for by Medicaid through the State Drug Utilization Database (SDUD). Due to lack of availability and missing data within some states, we only use data from eleven states for which we have complete data over the entire time period (California, Florida, Georgia, Indiana, Maryland, Minnesota, Missouri, New Jersey, New York, Tennessee, Wisconsin). For each of these 11 states, we identified 100 most frequently prescribed drugs according to correlation value between their quarterly prescription numbers and quarterly relative Google search volume of opioid-related keywords. The 100 most commonly filled prescriptions, based on NDC code, regardless of whether they were an opioid or not.

Quarterly information on opioid-related emergency department (ED) visits for the period April 2005 through March 2016 come from the Healthcare Cost and Utilization Project (HCUP) – State Emergency Department Database (SEDD). HCUP is a family of health care databases sponsored by the Agency for Healthcare Research and Quality (AHRQ) under the U.S. Department of Health and Human Services. The SEDD database in particular provides episode level information on all emergency visits to a hospital-affiliated ED that does not involve an admission to the hospital.

Statistical Analysis

The study was designed to determine the best fitting model incorporating Google Trends and/or drug utilization data from SDUD as predictors of opioid-related ED visits. To model the count data (number of ED visits), we used the negative binomial generalized linear model (nbGLM), a statistical model that is suitable for the modeling of count data. Because of its capability to address the overdispersion problem that commonly exists in real-world dataset, nbGLM has recently been widely applied to developing prediction model in public health research *(10,19)*. The prediction model is presented as equation (1).

Model (1) (both Google Trends and drug utilization data):

$$\ln(y(t+\Delta)_s) = w_{0,s} + \sum_{n=1}^{100} w_{n,s} \ln\left(D(t)_{n,s}\right) + \sum_{n=100}^{122} w_{n,s} \ln\left(G(t)_{n,s}\right) + \sum_{p=1}^{10} \alpha_p S_{p,s} + \varepsilon_t, \#(1)$$

where $y(t+\Delta)_s$ represents the number of ED visits of state s in time period $t+\Delta$. $D(t)_{n,s}$ denotes the utilization amount (number of unit) of the drug n in state s in time period t; $G(t)_{n,s}$ is the normalized composite Google search volume of the n-th keywords in state s in time period t; $S_{p,s}$ is the dummy variable representing a state; ε_t represents the white noise. We use the model to predict both the one-quarter ahead ($\Delta = 1$) ED visits and two-quarter ahead ($\Delta = 2$) ED visits. Log-transformation an established technique that rescales the actual observations in order to decrease the variability and make data conform to normality.

We adopted the Least Absolute Shrinkage and Selection Operator (LASSO) approach *(20)* to identify the subset of predictors that have the best predictive power among the list of search keywords and 100 most frequently used drugs captured in the SDUD for each state. The penalty coefficient (λ) that leads to the best fitting results was chosen for each prediction. The penalty factor enables LASSO to determine how many predictive variables that should be retained to maintain comparable prediction ability, which is advantageous than automated variable selection methods based on standard linear regression by stepwise selection or choosing features with the lowest p-values. In LASSO, cross-validation is used to choose the penalty factor to assure generalization. Smaller value of the cross-validation error with some penalty factor is expected to be better to express the generative model of the data. Since the association between predictors and the number of ED visits varies over time and across different states, we updated the predictor selection for each state. For time period $t + \Delta$ of each state, we used historical data from the third quarter of 2005 to time period t to predict the number of ED visits in time period $t + \Delta$. To train the model, we used the first eight years' data to estimate the parameters in the model, and then use the rest of the data to evaluate the prediction performance. To compare the predictabilities of Google Trends data and Medicaid drug utilization data, we evaluated the performance of two additional variants of the model:

Model 2 (only Google Trends data):

$$\ln(y(t+\Delta)_s) = w_{0,s} + \sum_{n=100}^{122} w_{n,s} \ln\left(G(t)_{n,s}\right) + \sum_{p=1}^{10} \alpha_p S_{p,s} + \varepsilon_t, \#(2)$$

Model 3 (only drug utilization data)

$$\ln(y(t)_s) = w_{0,s} + \sum_{n=1}^{100} w_{n,s} \ln\left(D(t)_{n,s}\right) + \sum_{p=1}^{10} \alpha_p S_{p,s} + \varepsilon_t. \#(3)$$

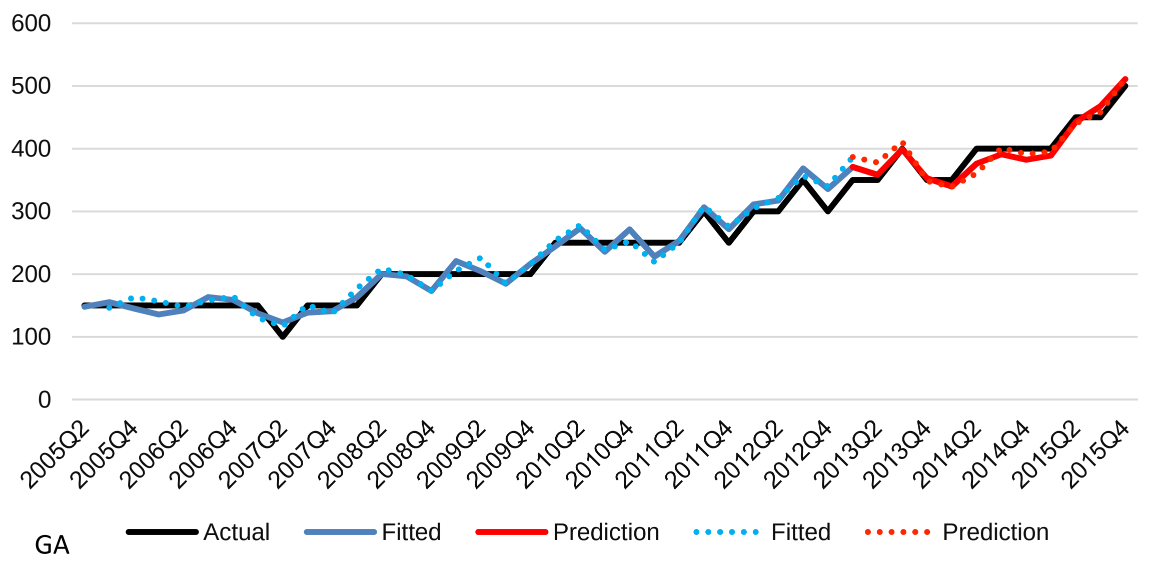

Supplementary Fig. 1. Results S1. One-quarter-ahead (solid) and two-quarter-ahead (dashed) predictions of the ED visits for Georgia (GA) based on Google search and drug use data.

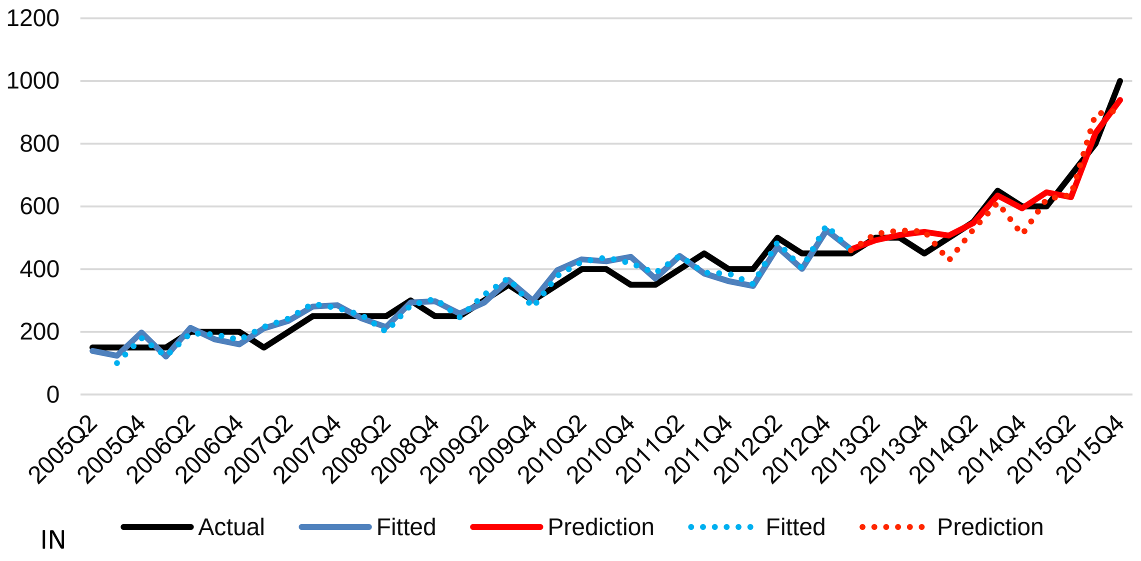

Supplementary Fig. 2. Results S2. One-quarter-ahead (solid) and two-quarter-ahead (dashed) predictions of the ED visits for Indiana (IN) based on Google search and drug use data.

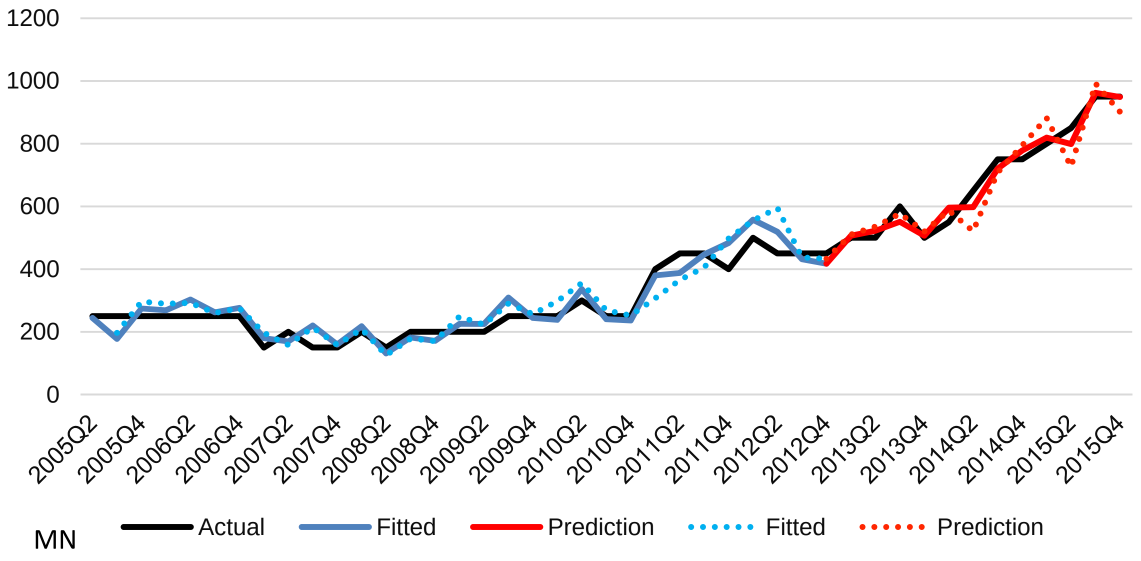

Supplementary Fig. 3. Results S3. One-quarter-ahead (solid) and two-quarter-ahead (dashed) predictions of the ED visits for Minnesota (MN) based on Google search and drug use data.

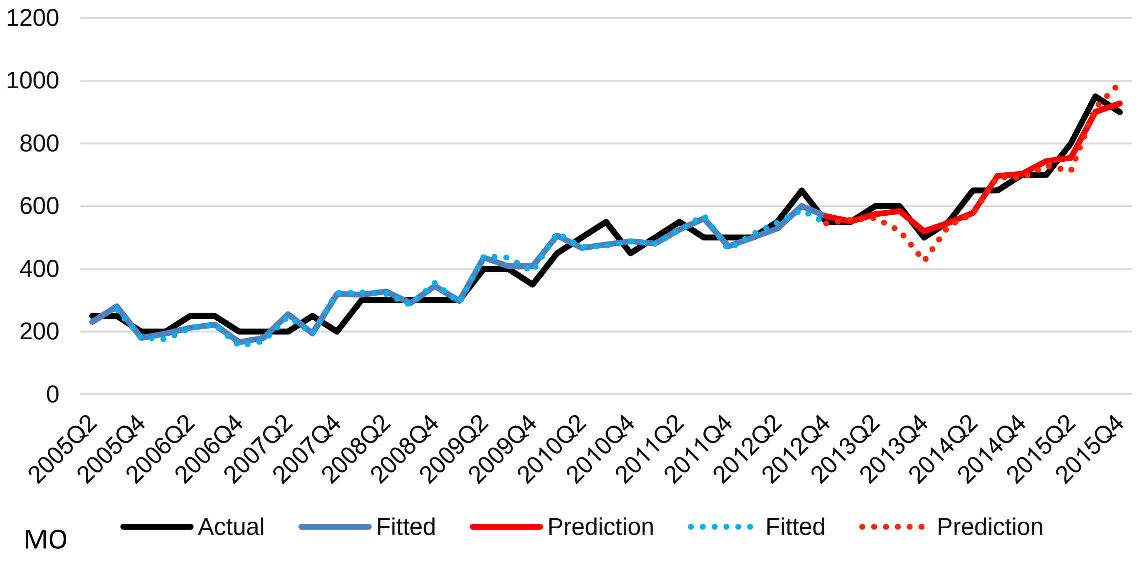

Supplementary Fig. 4. Results S4. One-quarter-ahead (solid) and two-quarter-ahead (dashed) predictions of the ED visits for Montana (MO) based on Google search and drug use data.

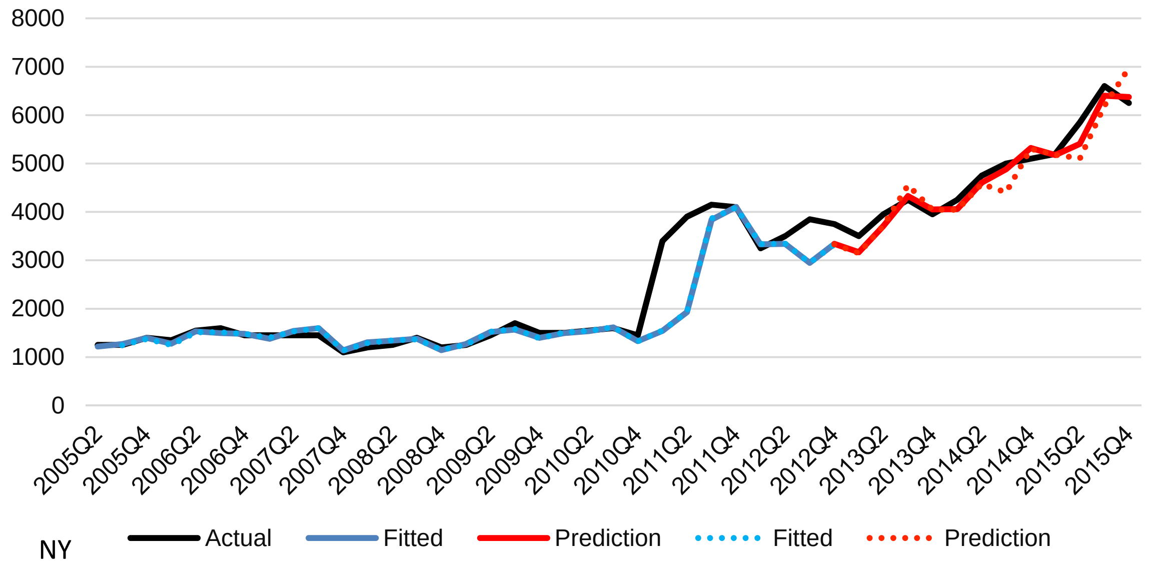

Supplementary Fig. 5. Results S5. One-quarter-ahead (solid) and two-quarter-ahead (dashed) predictions of the ED visits for New Jersey (NJ) based on Google search and drug use data.

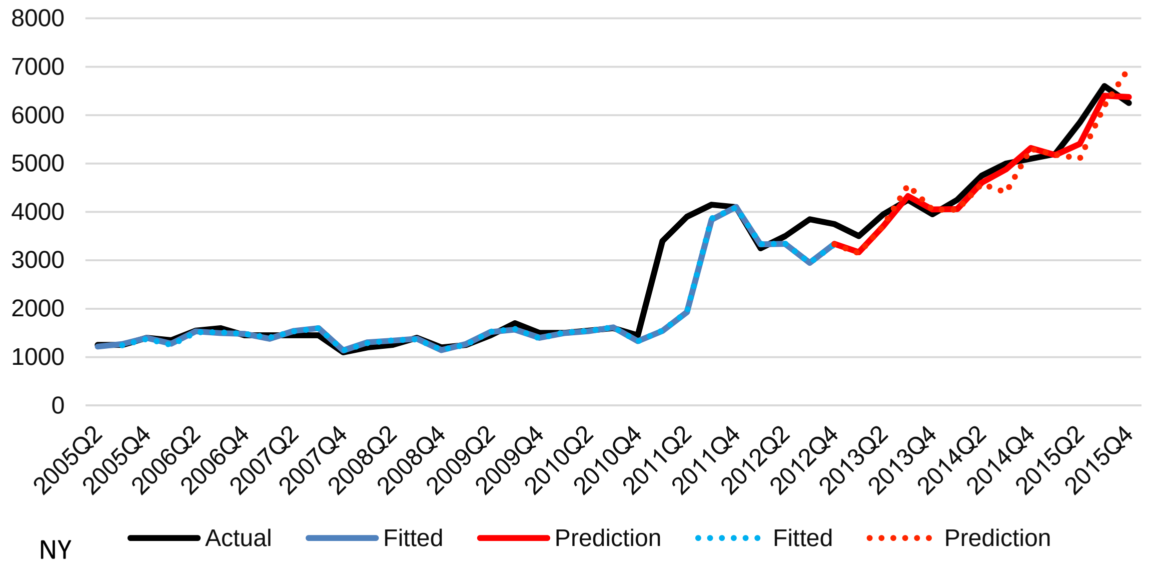

Supplementary Fig. 6. Results S6. One-quarter-ahead (solid) and two-quarter-ahead (dashed) predictions of the ED visits for New York (NY) based on Google search and drug use data.

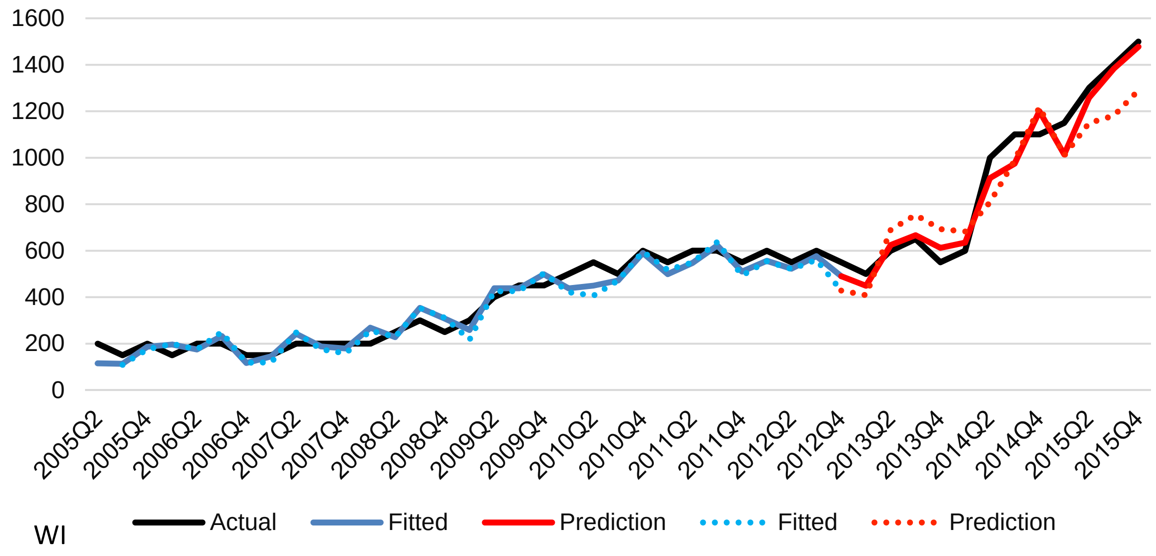

Supplementary Fig. 7. Results S7. One-quarter-ahead (solid) and two-quarter-ahead (dashed) predictions of the ED visits for Wisconsin (WI) based on Google search and drug use data.

Supplementary Table 1. 22 opioid-related terms that we used to collect the Google Trends data (also included in main manuscript file)

Fentanyl	Narcotic	Opioid analgesics	Opioid side effects
Heroin	Non opioid	Opioid antagonist	Opioid withdrawal
Hydrocodone	Opiates	Opioid conversion	Oxycodone
Methadone	Opioid	Opioid definition	Suboxone
Morphine	Opioid abuse	Opioid medications	Symptoms
Tramadol	What is opioid		

Supplementary Table 2. Opioid-related drugs and Google search terms for states with strongest association (also included in main manuscript file)

CA		FL		GA	
Top Drugs	Top Search Terms	Top Drugs	Top Search Terms	Top Drugs	Top Search Terms
LEVOCARNIT	fentanyl	LIDOCAINE -	fentanyl	OXYCODONE	opioid conversion
NOVOLOG PE	opioid withdrawal	TRAMADOL T	what is opioid	LEVOCARNIT	opioid
TRAMADOL T	non opioid	GUANFACINE	opioid abuse	PHOSPHA 25	suboxone
BACLOFEN T	methadone	GENOTROPIN	morphine	KENALOG-40	opiates
OXYCODONE	symptoms	ERBITUX	oxycodone	TRAMADOL H	fentanyl
INFED IRO	narcotic	BACITRACIN	opiates	NOVOLOG PE	opioid medications
ERBITUX	opioid abuse	OXYCODONE	heroin	VENTOLIN H	non opioid
OXYCODONE	opiates	MORPHINE	suboxone	ASPIRIN 5G	hydrocodone
TRAMADOL H	tramadol	MAGNESIUM	opioid	BACLOFEN T	methadone
MORPHINE	what is opioid	TRAMADOL H	opioid antagonist	ASPIRIN LO	morphine
**IN**		**MA**		**MN**	
Top Drugs	Top Search Terms	Top Drugs	Top Search Terms	Top Drugs	Top Search Terms
OXYCODONE	what is opioid	AMMONIUM L	non opioid	TRAMADOL T	non opioid
TRAMADOL H	oxycodone	OXYCODONE	opioid withdrawal	OXYCODONE	oxycodone
LEVOCARNIT	fentanyl	CALCIUM 50	fentanyl	NORETHINDR	opioid withdrawal
AZITHROMYC	heroin	EPIPEN JR	morphine	BACLOFEN T	fentanyl
KENALOG-40	non opioid	CARAFATE 1	heroin	NOVOLOG PE	oxycodone
MAGNESIUM	opioid withdrawal	CIPROFLOXA	oxycodone	CALCIUM 50	morphine
CIPROFLOX	opioid abuse	GENOTROPI	narcotic	ASPIRIN LO	methadone

A		N			
TRAMADOL T	morphine	TRAMADOL H	opioid abuse	KENALOG-40	narcotic
ASPIRIN 5G	opiates	MORPHINE	hydrocodone	TRUVADA 20	opioid abuse
CLINDAMY CI	methadone	LEVOCARNI T	suboxone	PREDNISOL O	hydrocodone
MO		NJ		NY	
Top Drugs	Top Search Terms	Top Drugs	Top Search Terms	Top Drugs	Top Search Terms
TRAMADOL 5	tramadol	TRAMADOL T	opiates	CLINDAMYC I	non opioid
LIQUITEARS	opiates	OXYCODON E	opioid	CHLORZOXA Z	fentanyl
ETHOSUXIM I	opioid	BUSPIRONE1	fentanyl	OXYCODON E	opioid withdrawal
FLUTICASO N	opioid withdrawal	DOCUSATE S	opioid withdrawal	BICILLIN L	opioid
TRAMADOL T	narcotic	MORPHINE	non opioid	TRAMADOL T	morphine
CALCIUM 50	fentanyl	EPIPEN JR	morphine	BUTALBITA L	suboxone
LEVOCARNI T	suboxone	ASPIRIN 5G	suboxone	LEVOCARNI T	hydrocodone
MORPHINE	morphine	KENALOG-40	morphine	MAGNESIUM	narcotic
ASPIRIN LO	hydrocodone	METRONIDA Z	hydrocodone	BACLOFEN T	oxycodone
NICOTINE 2	oxycodone	PHOSPHA 25	narcotic	CLINDAMYC I	tramadol
TN		WI			
Top Drugs	Top Search Terms	Top Drugs	Top Search Terms		
ERYPED 200	opioid	TRAMADOL H	fentanyl		
TRAMADOL H	tramadol	ASPIRIN 5G	opioid withdrawal		
MORPHINE	opioid conversion	EPIPEN EPI	non opioid		
CLINDAMY CI	morphine	MORPHINE	morphine		
KENALOG-40	opioid withdrawal	ARTIFICIAL	opiates		
EPIPEN JR	non opioid	EPIPEN JR.	narcotic		
LEVOCARNI T	narcotic	ANTIPYRINE	hydrocodone		

MAGNESIU M	fentanyl	OXYCODON E	opioid medications	
OXYCODON E	hydrocodone	LEVOCARNI T	oxycodone	
TRAMADOL T	oxycodone	10% PREMAS	tramadol	

Supplementary Table 3. One-quarter-ahead (and two-quarter ahead) prediction accuracy of different models across states using a mixed model, 2005-2015.

	Google Search Data		SDUD Rx Utilization Data		Both Google and Drug Data	
	R-squared	RMSE	R-squared	RMSE	R-squared	RMSE
CA	0.5554 (0.5669)	168.6195 (182.8960)	0.6285 (0.6143)	619.9324 (625.7042)	0.6719 (0.6917)	369.4448 (458.3274)
FL	0.6406 (0.6283)	219.1858 (197.3311)	0.7179 (0.7080)	111.1949 (93.9352)	0.7435 (0.7920)	105.9688 (89.0890)
GA	0.5394 (0.5107)	124.6534 (121.6111)	0.6419 (0.6419)	73.5516 (111.9991)	0.7383 (0.7616)	120.1866 (126.8049)
IN	0.6216 (0.6484)	167.4058 (163.2967)	0.7231 (0.7347)	134.3719 (192.2936)	0.7850 (0.7583)	44.6846 (58.3606)
MA	0.5470 (0.5441)	508.4880 (602.7853)	0.6729 (0.6502)	227.8877 (223.9812)	0.7775 (0.6974)	178.2275 (219.3894)
MN	0.6040 (0.5444)	86.6745 (173.2854)	0.7127 (0.7075)	118.9109 (192.5309)	0.7692 (0.7794)	107.4505 (124.0294)
MO	0.5979 (0.6060)	144.9457 (242.4950)	0.6661 (0.6548)	37.1436 (15.7437)	0.7841 (0.7165)	47.6939 (39.8260)
NJ	0.7106 (0.6536)	226.9919 (153.1767)	0.6699 (0.6859)	223.0611 (232.9615)	0.6837 (0.7321)	142.6544 (253.2262)
NY	0.5632 (0.5156)	349.3651 (314.4981)	0.7653 (0.7446)	340.0711 (419.3746)	0.7438 (0.6689)	321.0531 (256.8371)
TN	0.6418 (0.6299)	320.8542 (271.1485)	0.6748 (0.5965)	98.6367 (191.8528)	0.7022 (0.7131)	88.2356 (86.3717)
WI	0.5953 (0.5705)	264.0055 (325.1242)	0.6852 (0.7165)	134.2620 (192.7659)	0.7307 (0.6584)	136.3584 (94.8529)

Supplementary Table 4. One-quarter-ahead (and two-quarter ahead) prediction accuracy of different models across states using a pooling model, 2005-2015.

State	Google Search Data		SDUD Rx Utilization Data		Both Google and Drug Data	
	R-squared	RMSE	R-squared	RMSE	R-squared	RMSE
CA	0.5793 (0.5840)	196.5548 (184.5454)	0.6595 (0.5515)	600.0114 (641.8923)	0.7027 (0.6876)	364.1338 (456.0183)
FL	0.6531 (0.6088)	174.1758 (178.6484)	0.7349 (0.4182)	117.0109 (127.0257)	0.7851 (0.6719)	100.6812 (125.9055)
GA	0.5166 (0.4924)	134.3564 (126.0805)	0.6377 (0.4588)	70.8183 (95.5592)	0.7352 (0.5652)	127.3499 (118.2887)
IN	0.6476 (0.5856)	168.2415 (169.0808)	0.7335 (0.3963)	130.1494 (189.5271)	0.7729 (0.5566)	47.9801 (78.2179)
MA	0.5342 (0.6553)	534.4597 (585.3676)	0.6964 (0.5636)	249.7740 (212.9743)	0.7492 (0.7048)	211.7040 (220.5804)
MN	0.5746 (0.6630)	112.7012 (173.8752)	0.6969 (0.6018)	142.6623 (185.4659)	0.7808 (0.7179)	112.8283 (146.1750)
MO	0.6431 (0.6223)	187.1853 (241.0779)	0.6733 (0.5870)	37.6814 (20.9492)	0.7668 (0.6621)	62.7506 (36.8333)
NJ	0.6660 (0.5953)	197.3706 (153.5300)	0.6601 (0.6415)	220.3188 (219.7264)	0.7224 (0.7115)	169.5668 (254.4664)
NY	0.5546 (0.6571)	349.6786 (331.5456)	0.7414 (0.6492)	354.9234 (412.2609)	0.7171 (0.7030)	303.6311 (295.6557)
TN	0.6505 (0.5635)	300.5944 (298.6311)	0.6857 (0.6179)	120.3476 (165.9523)	0.7091 (0.6760)	61.2477 (79.6748)
WI	0.5998 (0.6302)	252.5109 (278.5431)	0.6901 (0.6018)	161.8558 (217.8328)	0.7133 (0.6905)	115.5622 (123.2220)

Supplementary Table 5. Forward stepwise regression model performance on one-quarter-ahead (and two-quarter ahead) prediction accuracy of across states, 2005-2015.

State	Google Search Data	SDUD Rx Utilization Data	Both Google and Drug Data
CA	0.8783(0.7865)	0.9144(0.8302)	0.9639(0.901)
FL	0.7995(0.7191)	0.845(0.7314)	0.9251(0.842)
GA	0.8666(0.6787)	0.8436(0.7437)	0.9117(0.8269)
IN	0.8572(0.8107)	0.8783(0.7523)	0.9489(0.8496)
MA	0.8436(0.751)	0.8861(0.7096)	0.9291(0.8215)
MN	0.9023(0.774)	0.8234(0.7823)	0.9619(0.8448)
MO	0.8567(0.7292)	0.875(0.7612)	0.9227(0.8137)
NJ	0.8624(0.7604)	0.8982(0.7821)	0.9506(0.8621)
NY	0.7589(0.7287)	0.8876(0.7185)	0.9401(0.8084)
TN	0.8584(0.742)	0.8967(0.7834)	0.9476(0.8298)
WI	0.8017(0.7535)	0.8931(0.7638)	0.9527(0.8197)

Supplementary Table 6. Backward stepwise regression model performance on one-quarter-ahead (and two-quarter ahead) prediction accuracy (R-squared) of across states, 2005-2015.

State	Google Search Data	SDUD Rx Utilization Data	Both Google and Drug Data
CA	0.8724(0.7785)	0.9056(0.8272)	0.9588(0.8936)
FL	0.8039(0.7255)	0.8488(0.7333)	0.9267(0.832)
GA	0.8627(0.6813)	0.8404(0.7433)	0.916(0.8216)
IN	0.8527(0.818)	0.8924(0.7592)	0.9355(0.8531)
MA	0.8547(0.7572)	0.894(0.7078)	0.9293(0.8137)
MN	0.8938(0.7736)	0.825(0.7855)	0.9595(0.8377)
MO	0.8566(0.7343)	0.8854(0.7698)	0.9215(0.8142)
NJ	0.8711(0.7682)	0.8983(0.78)	0.9521(0.8519)
NY	0.7554(0.7226)	0.8868(0.7289)	0.95(0.8037)
TN	0.8504(0.7474)	0.893(0.7777)	0.944(0.8318)
WI	0.8017(0.7515)	0.8967(0.7592)	0.9602(0.8289)
