# Supplementary material for: Internet search and medicaid prescription drug data as predictors of opioid emergency department visits
Source: NPJ Digit Med. 2021 Feb 11;4:21. doi: 10.1038/s41746-021-00392-w (PMC7878816; doi:10.1038/s41746-021-00392-w)
Supplement: Supplementary file 2 — Reporting Summary [file 41746_2021_392_MOESM2_ESM.pdf]

nature research

Corresponding author(s): Young

Last updated by author(s): Nov 4, 2020

Reporting Summary

Nature Research wishes to improve the reproducibility of the work that we publish. This form provides structure for consistency and transparency in reporting. For further information on Nature Research policies, see our Editorial Policies and the Editorial Policy Checklist.

Statistics

For all statistical analyses, confirm that the following items are present in the figure legend, table legend, main text, or Methods section.

n/a	Confirmed	
☒	☐	The exact sample size (n) for each experimental group/condition, given as a discrete number and unit of measurement
☒	☐	A statement on whether measurements were taken from distinct samples or whether the same sample was measured repeatedly
☒	☐	The statistical test(s) used AND whether they are one- or two-sided<br>*Only common tests should be described solely by name; describe more complex techniques in the Methods section.*
☐	☒	A description of all covariates tested
☐	☒	A description of any assumptions or corrections, such as tests of normality and adjustment for multiple comparisons
☐	☒	A full description of the statistical parameters including central tendency (e.g. means) or other basic estimates (e.g. regression coefficient) AND variation (e.g. standard deviation) or associated estimates of uncertainty (e.g. confidence intervals)
☒	☐	For null hypothesis testing, the test statistic (e.g. F, t, r) with confidence intervals, effect sizes, degrees of freedom and P value noted<br>*Give P values as exact values whenever suitable.*
☒	☐	For Bayesian analysis, information on the choice of priors and Markov chain Monte Carlo settings
☒	☐	For hierarchical and complex designs, identification of the appropriate level for tests and full reporting of outcomes
☒	☐	Estimates of effect sizes (e.g. Cohen's d, Pearson's r), indicating how they were calculated

Our web collection on statistics for biologists contains articles on many of the points above.

Software and code

Policy information about availability of computer code

Data collection	The data were from publicly available data. The Medicaid data can be accessed at https://data.medicaid.gov/. The Google trends data is provided by Google's free API. It can be accessed by the following Python package: http://pypi.org/project/pytrends/.
Data analysis	The codes are available at the following github site: https://github.com/jadonzhou/EDVisits

For manuscripts utilizing custom algorithms or software that are central to the research but not yet described in published literature, software must be made available to editors and reviewers. We strongly encourage code deposition in a community repository (e.g. GitHub). See the Nature Research guidelines for submitting code & software for further information.

Data

Policy information about availability of data

All manuscripts must include a data availability statement. This statement should provide the following information, where applicable:

- Accession codes, unique identifiers, or web links for publicly available datasets
- A list of figures that have associated raw data
- A description of any restrictions on data availability

The data used in this analysis are publicly available online through Medicaid and HCUP. Google data may be available upon request, pending confirmation from Google. Supplementary materials are provided to assist in replication by describing the complete models and math used for analysis.

Field-specific reporting

Please select the one below that is the best fit for your research. If you are not sure, read the appropriate sections before making your selection.

☐ Life sciences ☒ Behavioural & social sciences ☐ Ecological, evolutionary & environmental sciences

For a reference copy of the document with all sections, see nature.com/documents/nr-reporting-summary-flat.pdf

Behavioural & social sciences study design

All studies must disclose on these points even when the disclosure is negative.

Study description	*Briefly describe the study type including whether data are quantitative, qualitative, or mixed-methods (e.g. qualitative cross-sectional, quantitative experimental, mixed-methods case study).*
Research sample	From January 1, 2005 to Dec 31, 2015, we collected quarterly Google Trends data for 22 commonly used opioid-related internet search terms and phrases for all states from 2005 through 2015. The terms include opioid medications (e.g. fentanyl and hydrocodone), opioid recreational drugs (e.g. heroin), and general searches about opioids and overdose risk.
Sampling strategy	This was a convenience sample of Google data along with publicly available data on opioids. No power analysis was conducted.
Data collection	Data were collected and retrieved online.
Timing	Data included the range from January 1, 2005 to Dec 31, 2015. We collected quarterly data ffrom 2005 through 2015.
Data exclusions	We included the eleven states with complete data for each year (California, Florida, Georgia, Indiana, Maryland, Minnesota, Missouri, New Jersey, New York, Tennessee, Wisconsin) in the final analysis.
Non-participation	N/A
Randomization	N/A

Reporting for specific materials, systems and methods

We require information from authors about some types of materials, experimental systems and methods used in many studies. Here, indicate whether each material, system or method listed is relevant to your study. If you are not sure if a list item applies to your research, read the appropriate section before selecting a response.

Materials & experimental systems

n/a	Involved in the study	
☒	☐	Antibodies
☒	☐	Eukaryotic cell lines
☒	☐	Palaeontology and archaeology
☒	☐	Animals and other organisms
☒	☐	Human research participants
☒	☐	Clinical data
☒	☐	Dual use research of concern

Methods

n/a	Involved in the study	
☒	☐	ChIP-seq
☒	☐	Flow cytometry
☒	☐	MRI-based neuroimaging
